# Impact of an inpatient multidisciplinary glucose control management program

**DOI:** 10.20945/2359-3997000000071

**Published:** 2018-10-01

**Authors:** Denise P Momesso, Rubens Carmo Costa, João Luiz Ferreira Costa, Felipe Saddy, Ayla Mesquita, Marcela Calomeni, Claudia dos Santos Silva, Jacqueline Farret, Mariana Leon Vasques, Aline G. Santos, Ana Paula Vieira Cabral, Dayane Ribeiro, Luciana Reis, Maria de Fátima M. Muino, Roberta Santiago Vitorino, Claudio Amorim Monteiro, Evandro Tinoco, Andre Volschan

**Affiliations:** 1 Hospital Pró-Cardíaco Hospital Pró-Cardíaco Rio de Janeiro RJ Brasil Hospital Pró-Cardíaco, Rio de Janeiro, RJ, Brasil

**Keywords:** Hyperglycemia, hypoglycemia, inpatient glucose control, diabetes mellitus, systems, point-of-care

## Abstract

**Objective:**

Glycemic control has been increasingly recognized as a critical element in inpatient care, but optimal management of blood glucose in the hospital setting remains challenging. The aims of this study were to describe and evaluate the impact of the implementation of an inpatient multidisciplinary glucose control management program on glucose control in hospitalized patients.

**Materials and methods:**

Retrospective analysis of medical records and glucose monitoring data obtained by point- of-care testing (POCT) in hospitalized patients before (May 2014) and after (June 2015 and May 2017) the implementation of the program.

**Results:**

We analyzed 6888, 7290, and 7669 POCTs from 389, 545, and 475 patients in May 2014, June 2015, and May 2017, respectively. Hyperglycemia (≥ 180 mg/ dL) occurred in 23.5%, 19.6%, and 19.3% POCTs in May 2014, June 2015, and May/2017, respectively (p < 0.001), while severe hyperglycemia (≥ 300 mg/dL) was observed in 2.5%, 2.2%, and 1.8% of them, respectively (p = 0.003). Hyperglycemia (≥ 180 mg/dL) reduced significantly from May 2014 to June 2015 (16.3%, p < 0.001) and from May 2014 to May 2017 (178%, p < 0.001). No significant changes occurred in hypoglycemic parameters.

**Conclusions:**

The implementation of an inpatient multidisciplinary glucose control management program led to significant reductions in hyperglycemic events. The key elements for this achievement were the development of institutional inpatient glycemic control protocols, establishment of a multidisciplinary team, and continuing educational programs for hospital personnel. Altogether, these actions resulted in improvements in care processes, patient safety, and clinical outcomes of hospitalized patients.

## INTRODUCTION

Glycemic control has been increasingly recognized as a critical element in inpatient care (1-7). Several lines of evidence corroborate the concept that both hyperglycemia and hypoglycemia are associated with adverse outcomes to the patient. Hyperglycemia may occur in hospitalized patients with known diabetes or acutely ill individuals with previously normal glucose tolerance (“stress hyperglycemia”) due to increased circulating counterregulatory hormones in response to stress. Irrespective of the cause, hyperglycemia on an inpatient setting is an independent marker of increased morbidity and mortality. During treatment of hyperglycemia, a major concern is the occurrence of hypoglycemia, which is also an independent risk factor for poor clinical outcomes (8-15). Therefore, great emphasis has been placed on optimizing the treatment of hospitalized patients with diabetes and hyperglycemia.

Based on data from multiples studies and clinical trials, the management of hyperglycemia in a hospital setting has recently evolved (16-23). Current consensus statements from the American Diabetes Association, American Association of Clinical Endocrinologists, Endocrine Society, and Brazilian Diabetes Association have recommended therapy of critically ill patients with persistent hyperglycemia, starting at a blood glucose level of 180 mg/dL; once insulin is started, the therapeutic glucose target should be within the 140180 mg/dL range (1-6). In noncritical care patients, the recommended values are < 140 mg/dL for fasting glucose and < 180 mg/dL for random glucose, and the preferable regimen is the use of a basal insulin along with premeal and supplemental insulin, instead of sliding scale insulin. Optimal glycemic control also includes the prevention of hypoglycemia (1-6,24-26). Reducing the variability in glucose levels may also be important in improving outcomes (27,28).

Despite existing evidence, the optimal glucose management in a hospital setting remains challenging, as the achievement of improved glycemic control in a hospital setting meets numerous obstacles. In this scenario, the American College of Endocrinology, the American Diabetes Association, and the Brazilian Diabetes Society have released calls to action outlining strategies for a successful implementation of inpatient glucose control management programs (2,5,7). System- based key issues outlined included the need for the development and evaluation of: 1) clinical protocols to guide management, clinical decisions, and prescriptions; 2) multidisciplinary glucose control management teams; and 3) provider-delivered educational programs to improve knowledge and address barriers to achieving glycemic control (2,5,7,29,30).

The aims of this study were to describe and evaluate the impact of the implementation of a multidisciplinary glucose control management program for hospitalized patients. The study was conducted to determine if such program would improve inpatient safety by reducing the number of hyperglycemic events.

## MATERIALS AND METHODS

The study was conducted at the *Pró-Cardíaco Hospital,* a 99-bed, tertiary-care, medical and surgical center located in Rio de Janeiro, Brazil. The medical center comprises an emergency room, two adult intensive care units (ICU), one coronary care unit, one surgical intensive care unit, one surgical semi-intensive care unit, three clinical semi-intensive units, one oncology care unit, and one day clinic. The hospital has an extensive referral network and includes outpatient specialty treatment and imaging centers, clinics, and rehabilitation centers.

Since 2001, the ICUs of the hospital have developed a protocol of insulin treatment for critically ill patients. In 2012, we introduced a hospital-wide inpatient multidisciplinary glucose control management program (MGCP) to facilitate the development of uniform glucose management policies and staff education based on current clinical practice guidelines. The main hallmarks of this program were the development of an Institutional inpatient glycemic control protocols in January 2012, and the establishment of a multidisciplinary glycemic control team in June 2014.

### Institutional inpatient glycemic control protocols

In 2012, institutional glycemic control protocols were developed in an effort to improve and standardize the glycemic control of hospitalized patients. These protocols are aligned with international and local recommendations (2-6) and are frequently revised and updated based on these recommendations and local assistance requirements.

According to the institutional protocol, blood glucose levels of all critical and noncritical care patients admitted to the hospital are monitored using point- of-care testing (POCT). Glucose monitoring may be suspended 72 hours after admission of noncritically ill patients and in those without diabetes or current illness, or not using medications associated with hyperglycemia or hypoglycemia. Monitoring may also be suspended in patients whose blood glucose measurements have been within the normal range for 72 hours. All results obtained by POCT are downloaded directly from the glucometer (Precision^®^, Abbott Diabetes Care Inc., Alameda, CA, USA) to the patient's electronic medical records. In case of glucose measurements ≤ 40 mg/dL or ≥ 300 mg/dL, a direct notification is sent via e-mail from the medical record to the endocrinologist in charge of the patient.

Insulin therapy is the method of choice for glycemic control in hospitalized patients with hyperglycemia. The institutional protocol recommends the discontinuation of antidiabetic drugs for most patients upon hospital admission for acute illness. Patients with type 1 or 2 diabetes receiving insulin as multiple daily injections require treatment with basal-bolus insulin regimens, and their insulin doses are modified according to the patient's clinical status.

In noncritical care patients, the glycemic targets set by the institutional protocol are < 140 mg/dL before meals and < 180 mg/dL for random glucose measurements. In patients with terminal illness and/ or limited life expectancy, the glycemic target is < 180 mg/dL. In noncritical care patients, POCTs are performed based on the timing of the meals: before meals in patients receiving an oral diet, every 6 hours in those with continuous enteral or parenteral nutrition, and every 4 hours in patients not receiving diet. Patients with hyperglycemia (glucose level ≥ 200 mg/dL) undergo more frequent glucose measurements for detection and treatment of hyperglycemia, prevention of hypoglycemia following supplemental insulin administration, and prevention of glycemic variability. The protocol recommends a basal-bolus insulin regimen, including a basal component with a long- acting insulin analogue (glargine or detemir) or intermediate-acting insulin (NPH) once or twice daily, and a bolus component with ultra-rapid-acting insulin (lispro) administered according to meals and supplemental doses according to glycemic levels. Ultrarapid insulin analogues are the insulin of choice for the bolus component of the regimen at our institution, based on evidence in the literature showing better glycemic control in hospitalized patients with this type of insulin when compared with regular insulin, with a lower number of hypoglycemic episodes (24). The total dose of insulin administered is individualized and based on the patient's previous insulin regimen, glycemic levels, total body weight, clinical status, and nutritional therapy. Basal insulin is administered to all patients with previous insulin regimens and in those with sustained hyperglycemia. In insulin-naive patients, the recommended initial total daily insulin dose is 0.20.5 U/kg/day, with approximately 50% of the dose administered as basal insulin (preferably glargine) and the remainder as bolus insulin. Bolus insulin contemplates the patient's diet and carbohydrate intake, as follows: bolus insulin before meals in patients on an oral diet, every 6 hours in those on continuous enteral or parenteral nutrition therapy, and 3 to 4 times a day before meals in patients receiving cyclic enteral nutrition. The supplemental component of bolus insulin is administered according to the patient's glucose level. Our institution has five different supplemental insulin regimens: (i) usual insulin dose; (ii) reduced insulin dose, recommended for patients at risk for hypoglycemia; (iii) increased insulin dose, recommended for patients with insulin resistance; (iv) a regimen for patients with terminal illness and/or limited life expectancy; and (v) a regimen for patients with no oral or enteral nutritional therapy (fasting).

For critical care patients, blood glucose levels are measured at one-hour intervals. Insulin therapy is started in critically ill patients with sustained hyperglycemia, defined as at least two glucose measurements ≥ 180 mg/dL, using continuous intravenous regular insulin infusion. In patients with continuous intravenous insulin infusion, the glycemic targets are 140-180 mg/dL, and glucose levels < 100 mg/dL should be avoided. Insulin infusion is adjusted every hour according to glucose levels. The continuous intravenous insulin infusion protocol used at the institution was adapted from the Yale Insulin Infusion Protocol for critically ill patients, as previously described (23).

### Multidisciplinary glycemic control team

A multidisciplinary glycemic control team was created at our institution in June 2014. The main goal of its implementation was to develop a centralized multidisciplinary team to address barriers to achieving glycemic control in the hospital setting. The team is chaired by an endocrinologist and includes physicians (endocrinologist, intensivists, hospitalists, and house staff), nurse practitioners, pharmacists, dietitians, and POCT/laboratory medicine specialists. The team aims at promoting the correct implementation of protocols for management of hyperglycemia and hypoglycemia, educating physicians and nurses on the proper use of these protocols, performing continuous education of health care professionals, promoting clinical decision aids, and surveilling performance measures for quality improvement. The members of the team deliberate on regular monthly meetings and on a daily basis during continuous patient care.

### Education programs for hospital personnel

The educational programs for hospital personnel are delivered on a regular basis and include the participation of nurses, house staff, physicians, pharmacists, and dietitians. Educational sessions are often offered in the hospital at different time periods to ensure delivery to as many staff members as possible. All staff members (physicians, nurses, pharmacists, and dietitians) are exposed to the educational program upon joining the hospital staff. Regular educational sessions are delivered at different hospital units (a total of 10 units) at a maximum interval of 6 months. Once weekly, the endocrinologist in charge delivers educational orientation to staff members during patient care (onsite training).

The main aspects outlined in these educational programs include the impact of inpatient glycemic control on patient care, introduction and reinforcement of protocols for management of hyperglycemia and hypoglycemia, information about patients at risk for hypoglycemia and hyperglycemia, identification of signs of hypoglycemia, characteristics of the different types of insulin and administration routes (intravenous or subcutaneous for basal insulin administration, prandial or correction doses), a review of insulin requirements during health and illness, inpatient use of antihyperglycemic agents, the influence of diet, the importance of respecting the appropriate time of glucose measurement, and proper documentation of patient treatment.

### Glucose monitoring by point-of-care testing

Glucose monitoring by POCT was performed with the glucometers Precision Xceed Pro (PXP)^®^ and FreeStyle Precision Pro (FSPP)^®^ (Abbott Diabetes Care Inc., Alameda, CA, USA). In noncritical patients, glycemic measurements with POCT used capillary blood samples obtained by fingertip puncture after local hygiene, while in critical patients, venous or arterial blood samples were used instead. The same type of blood source was used in each patient according to his or her clinical status. Areas with edema, lesions, hypoperfusion, and/ or venous infusion routes with continuous infusion of solutions were avoided during blood drawing.

The glucometers underwent continuous quality control. All devices were calibrated every 24 hours with high and low glucose control samples; when calibration was not performed in 24 hours, the glucometer was automatically blocked from use or for POCT. All glucometers underwent a harmonization process every 6 months, consisting on a comparison of the results obtained by POCT with those obtained by the laboratory (Dimension^®^, Siemens Healthcare Diagnostics, Deerfield, IL, USA). During the study, the variation coefficient between the results obtained by the POCT and those by the laboratory was < 10.78% in all glucometers, which is aligned with standards of care (31).

### Glycemic control quality indicators

Since the MGCP implementation, quality indicators of glycemic control, hypoglycemia, and hyperglycemia were monthly assessed using data from the POCTs. All results obtained by POCT were electronically downloaded directly from Abbott's Precision^®^ glucometer to the software using the Abbott Precision Web System (Abbott Diabetes Care Inc., Alameda, CA, USA) and to the patient's electronic medical records, providing accurate data for the indicators. Calculation of the rates of hyperglycemia and hypoglycemia were as follows: (i) hyperglycemia (≥ 180 mg/dL) as the number of glucose measurements by POCT ≥ 180 mg/dL (numerator) divided by the total number of glucose measurements by POCT performed in that given period (denominator), (ii) severe hyperglycemia (≥ 300 mg/dL) as the number of glucose measurements by POCT ≥ 300 mg/dL (numerator) divided by the total number of glucose measurements by POCT performed in the period (denominator), (iii) hypoglycemia (≤ 70 mg/dL) as the number of glycemic measurement by POCT ≤ 70 mg/dL (numerator) divided by the total number of glycemic measurement by POCT performed in the period (denominator), (iv) severe hypoglycemia (≤ 40 mg/dL) as the number of glycemic measurement by POCT ≤ 40 mg/dL (numerator) divided by the total number of glycemic measurement by POCT performed in the period (denominator).

Adherence to the institutional inpatient glycemic protocols was measured regularly by a revision of the patient's prescriptions and medical records.

### Data collection

We performed a retrospective analysis of the medical records of the patients admitted to the hospital. We analyzed the data obtained in May 2014, before the MGCP implementation, and in June 2015 and May 2017, after the MGCP implementation.

The inclusion criteria were all critical and noncritical patients admitted to the hospital, aged ≥ 18 years, who had blood glucose measured by POCT by Abbott's Precision^®^ glucometer according to the institutional protocol, and a length of stay ≥ 2 days. The exclusion criteria were age < 18 years, length of stay shorter than 2 days, and admissions limited to the emergency room or to the day-clinic unit. We also excluded the results of POCT glucose monitoring obtained during surgical procedures.

We analyzed the quality indicators of glycemic control and the results of glucose monitoring obtained by POCT before the implementation of the MGCP in May 2014 and after the implementation of the MGCP in June 2015 and May 2017. Based on blood glucose levels, the patients were characterized as having hyperglycemia (≥ 180 mg/dL), severe hyperglycemia (≥ 300 mg/dL), hypoglycemia (≤ 70 mg/dL), or severe hypoglycemia (≤ 40 mg/dL). The rates of hyperglycemia (≥ 180 mg/dL), severe hyperglycemia (≥ 300 mg/dL), hypoglycemia (≤ 70 mg/dL), and severe hypoglycemia (≤ 40 mg/dL) were calculated as described above.

Adherence to the institutional inpatient glycemic protocols was also analyzed by a revision of the patients’ prescriptions and medical records in May 2014, June 2015, and May 2017. A prescription was considered to be not compliant to the institutional protocols if inadequate to the diet or clinical scenario. Inadequacy with the diet was present when the prescription was not conformed with the type of diet (*i.e.,* oral, enteral, and parenteral diets, or fasting), not coordinated with the POCT, or when insulin was not administered around mealtime. Inadequacy with the clinical scenario occurred when the insulin prescribed was not suitable for the patient's clinical status according to the institutional protocol (*i.e*., intravenous insulin for critical patients, subcutaneous insulin for noncritical patients, basal-bolus insulin regimen for noncritical patients with sustained hyperglycemia and/or previous use of a basal-bolus insulin regimen, or supplemental subcutaneous insulin for noncritical patients in accordance to patients characteristics (usual insulin dose, risk of hypoglycemia, insulin resistance, terminal illness, and/or limited life expectancy).

The local institutional ethics committee approved the study.

### Statistical analysis

Continuous data are presented as mean and standard deviation values or median values and range. Comparisons of categorical variables were performed with the Fisher's exact or chi-square test, while continuous variables were compared using Student's t test.

The statistical analyses were performed with the software programs SPSS, version 20 (SPSS Inc., Chicago, IL, USA), Minitab 16, and Excel Office 2010. Statistical significance was set at p < 0.05.

## RESULTS

The clinical characteristics of the hospitalized patients undergoing glucose monitoring in May 2014, June 2015, and May 2017 are described in Table 1. The groups had comparable baseline clinical characteristics except for age, which differed among the groups.

**Table 1:** Baseline clinical characteristics of hospitalized patients with glucose measured by point-of-care testing (POCT)

		May 2014	June 2015	May 2017	p value
Number of patients undergoing POCT		389	545	475	
Age (years)		72.7 ± 16.6	73.0 ± 16.2	75.6 ± 14.6	0.04*
Admission					
	Clinical patients	80.8%	83.9%	78.5%	0.086
	Surgical patients	19.2%	16.1%	21.5%	
Patients’ characteristics at admission					
	Critical care	10.4%	13.4%	9.7%	0.141
	Noncritical care	89.6%	86.6%	90.3%	
	Diabetes mellitus	33.7%	30.4%	35.1%	0.176
Rate of hyperglycemia (≥ 180 mg/dL) on admission		22.1%	18.9%	19.6%	0.465
Length of stay (days)		5 (2-919)	5 (2-919)	5 (2-967)	0.98
Intravenous insulin protocol		7.3%	8.8%	6.5%	0.371

Data are expressed as mean ± standard deviation, mean (range), or percentage.

* Statistically significant (p < 0.05).

We analyzed 6888, 7290, and 7669 POCTs from 389, 545, and 475 patients in May 2014, June 2015, and May 2017, respectively. The mean number of glucose measurements per patient was 2.39 ± 1.96, 2.39 ± 2.26, and 3.64 ± 2.76 in May 2014, June 2015, and May 2017, respectively. There was a significant increase in glucose monitoring from May 2014 to May 2017 (p = 0.007), but no differences between May 2014 and June 2015 (p = 0.99).

**Figure 1:**
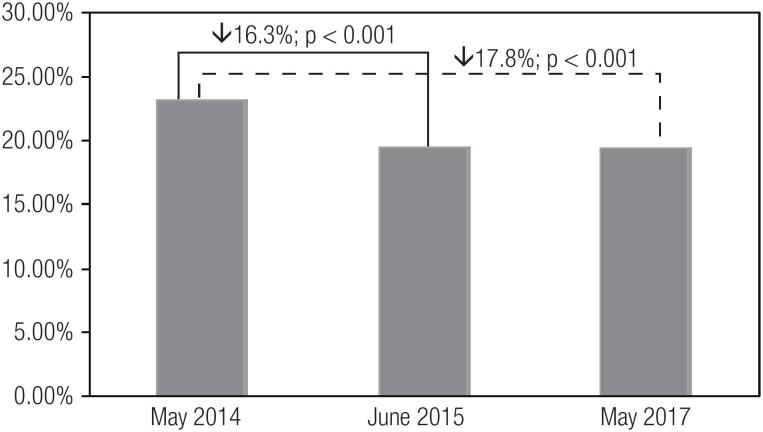
Rates of hyperglycemia (≥ 180 mg/dL) in hospitalized patients in May 2014, June 2015, and May 2017.

**Figure 2:**
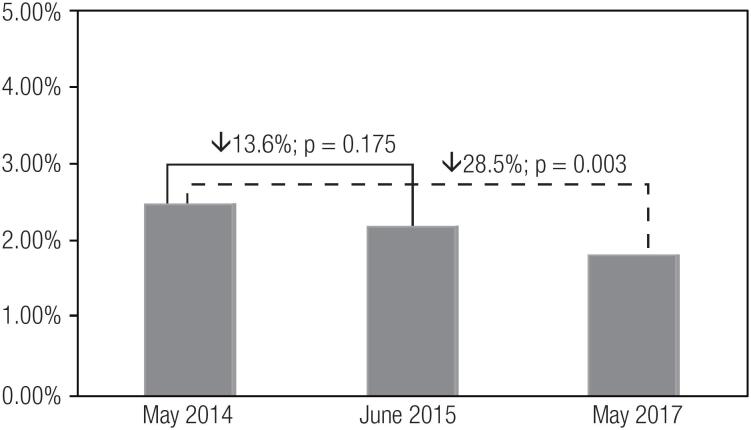
Rates of severe hyperglycemia (≥ 300 mg/dL) among hospitalized patients in May 2014, June 2015, and May 2017.

Table 2 describes the quality indicators of inpatient glycemic control. In May 2014, June 2015, and May 2017, the rates were 23.5%, 19.6%, and 19.3%, respectively, for hyperglycemia (≥ 180 mg/dL; p < 0.001), and 2.5%, 2.2%, and 1.8%, respectively, for severe hyperglycemia (≥ 300 mg/dL; p = 0.003). The rates of hyperglycemia (≥ 180 mg/dL) reduced significantly from May 2014 to June 2015 (16.3%, p < 0.001) and from May 2014 to May 2017 (17.8%, p < 0.001). Similarly, the rates of severe hyperglycemia (≥ 300 mg/dL) reduced statistically significantly from May 2014 to May 2017 (28.5%, p = 0.003), but were non-statistically significant between May 2014 and June 2015 (13.6%, p = 0.175) (Figures 1 and 2).

**Table 2:** Quality indicators of inpatient glycemic control

	May 2014	June 2015	May 2017	p value
Number of patients undergoing POCT	389	545	475	–
Number of glucose readings with POCT	6888	7290	7669	–
Glucose level - mean (SD) (mg/dL)	158.9 ± 60.7	150.5 ± 59.1	150.3 ± 57.8	< 0.001*
Glucose level - median (IQR) (mg/dL)	147.0 (118-188)	138.0 (1120-177)	137.0 (109-178)	< 0.001*
Rate of severe hypoglycemia (≤ 40 mg/dL)	0.2% (n = 12)	0.1% (n = 7)	0.1% (n = 9)	0.336
Rate of hypoglycemia (≤ 70 mg/dL)	0.9% (n = 65)	1.8% (n = 129)	1.0% (n = 79)	0.710
Rate of hyperglycemia (≥ 180 mg/dL)	23.5% (n = 1620)	19.6% (n = 1428)	19.3% (n = 1521)	< 0.001*
Rate of severe hyperglycemia (≥ 300 mg/dL)	2.5% (n = 175)	2.2% (n = 160)	1.8% (n = 143)	0.003*

SD: standard deviation; IQR: interquartile range; POCT: point-of-care testing.

* Statistically significant (p < 0.05).

There was no statistically significant change in hypoglycemic parameters over time (Table 2). Rates of hypoglycemia (≤ 70 mg/dL) were 0.9%, 1.8%, and 1.0% in May 2014, June 2015, and May 2017, respectively (p = 0.710), while the rates of severe hypoglycemia (≤ 40 mg/dL) showed a non-statistically significant decrease of 34.3% from May 2014 to May 2017 (p = 0.336).

Table 3 describes the rates of adherence to the institutional inpatient glycemic protocols. Adherence to the protocol improved over time. The proportion of prescriptions not compliant with the institutional protocols decreased from 34.2% in May 2014 to 10.1% in June 2015, and 7.5% in May 2017 (p < 0.001). We found significant decreases in diet and clinical scenario inadequacies over time from May 2014 to May 2017.

**Table 3:** Rates of adherence to the institutional inpatient glycemic protocols

	May 2014	June 2015	May 2017	p value
Prescriptions not compliant with the institutional protocols	34.2%	10.1%	7.5%	< 0.001*
Noncompliance with clinical scenario	20.0%	6.3%	5.2%	0.026*
Noncompliance with diet	17.4%	3.8%	2.5%	0.006*

Data are expressed as percentage.

* Statistically significant (p < 0.05).

## DISCUSSION

The implementation of an inpatient multidisciplinary glucose control management program (MGCP) had a positive impact on glycemic control in hospitalized patients at our center. We observed that the key elements for this achievement were the implementation of institutional inpatient glycemic control protocols, establishment of a multidisciplinary glycemic control team, and continuous educational programs for hospital personnel. Altogether, these actions resulted in a significant reduction in hyperglycemic events and improved safety among inpatients.

The development of institutional inpatient glycemic control protocols was important in guiding initial management, clinical decisions, and prescriptions at our center. With the protocols, we were able to standardize our policies and the patients’ glycemic control. Since the implementation of the institutional protocols, different educational programs were delivered for hospital staff training. These protocols need to be constantly reevaluated and updated based on newly available evidence in the literature and on local demands of patient care. Other centers have described similar improvements in clinical outcomes with the adoption of insulin protocols for glucose management in critical and noncritical patients (17,18,23,29,30,31-33).

Considered alone, the implementation of the institutional inpatient glycemic control was probably not enough to improve the process of care. The protocol was implemented in January 2012, and in May 2014, we observed that a great proportion of the prescriptions were still not compliant to the protocol. We then hypothesized that the staff education programs should be optimized and further actions should be taken, including the implementation of a multidisciplinary glycemic control team and the dissemination of quality indicators of glucose control. Indeed, we found that the implementation of hospital-wide glucose policies was best facilitated by targeted educational programs and clinical decision support infrastructure to facilitate acceptance by the hospital personnel. We then observed a significantly increased adherence to the institutional inpatient glycemic control protocols over time, accompanied by improved quality indicators of glycemic control in June 2015 and May 2017.

The establishment of a centralized multidisciplinary glycemic control team was a core and critical element in the development of our inpatient glucose management program. Through regularly scheduled monthly meetings and a culture of collaboration and teamwork, the members of the team promoted the implementation of protocols, education interventions, clinical decision aids, performance measures, and quality indicators of I glycemic control across continuous inpatient care. In fact, I we observed significant reductions of 17.8% in the rate of hyperglycemia (≥ 180 mg/dL) and 28.5% in the rate of severe hyperglycemia (≥ 300 mg/dL) from May 2014 to May 2017, before and after the implementation of the multidisciplinary glycemic control team, respectively. These reductions in hyperglycemic events were already observed one year before (in June 2015), and improved even further in May 2017, suggesting a continuous improvement in patient care and quality outcomes.

Our educational programs focused on the major challenges to optimal glucose management. Similar to other centers, the main obstacles we encountered included unanticipated nutritional changes, poor coordination of the POCT with the administration of insulin around mealtime, unanticipated changes in clinical status or medications, use of medications associated with increased insulin resistance (such as glucocorticoids, often in variable and changing doses), failure by clinicians of making adjustments in glycemic therapy based on daily blood glucose patterns, prolonged use of sliding scale insulin as monotherapy, multiple system/organizational barriers such as lack of communication and/or deficient knowledge of diabetes management among providers and caregivers (7,29,30,32,33). Notably, we demonstrated that a collaborative work of the nurses, dietitians, and physicians reduced the inadequacy of the prescription with the type of diet and improved the coordination of POCT and administration of insulin around mealtime.

The impact of the MGCP on hypoglycemic events in inpatients was less established. We observed a nonsignificant 34.3% reduction in the rate of severe hypoglycemia (≤ 40 mg/dL), which might be due to the low rate of such event at our center. The rates of hypoglycemia (≤ 70 mg/dL) were similar over time. Hypoglycemia is a possible unwanted consequence of improved control of hyperglycemia and may be associated with increased morbidity (3-5,21). Therefore, our results of reduced hyperglycemia without increased hypoglycemic events demonstrate that our institutional protocols were safe. Indeed, it has been demonstrated that the implementation of standardized insulin order sets with less strict glycemic targets and frequent glucose monitorization are associated with better glycemic control and produce expected benefits in terms of patient safety across different hospitals (18,23,25,29,30,32,33).

Our study has limitations inherent to its retrospective, nonrandomized design and the absence of a concurrent control group. This study was intended to evaluate intermediary outcomes as a quality improvement for hospitalized patients, and we did not evaluate morbidity, mortality, or other important clinical outcome data other than the rates of hyperglycemia and hypoglycemia. Another limitation regarding the analysis of the glycemic data was the potential for an increased type I error (*i.e.,* a false-positive result) due to clustering of POCT values within patients and increased monitoring frequency upon observation of a hyperglycemic event. Indeed, the number of glucose monitoring tests among patients with normal glucose values may affect the proportion of abnormal values. Nevertheless, according to our institutional protocol, patients with hyperglycemia have more frequent POCT than those with normal glucose values.

Finally, despite the decrease in hyperglycemia rates, they still require further reduction, and efforts will be made for this purpose. Despite the fact that our quality indicators of glycemic control seem to be aligned with those of other hospitals, hyperglycemia in hospitalized patients is still frequently observed (34). The creation of a national benchmarking process would be important for the development of best practices and improved management of inpatient hyperglycemia (35).

In conclusion, the implementation of an inpatient multidisciplinary glucose control management program at our center was associated with improved care process and clinical outcomes, demonstrated by continued reductions in rates of hyperglycemic events. The key elements for these achievements were the development of institutional inpatient glycemic control protocols, establishment of a multidisciplinary team, and continuing educational programs for hospital personnel. Therefore, our results suggest that an inpatient multidisciplinary glucose control management program increased the awareness of the value of treating hyperglycemia in hospitalized patients, representing an important feature for inpatient safety and quality improvement.
